# Global research trends on macrophage polarization in osteoarthritis: a bibliometric analysis

**DOI:** 10.3389/fimmu.2025.1659938

**Published:** 2025-10-17

**Authors:** Abdulaziz S. Bamahel, Jia Liu, Zhuoyu Zhang, Yizhuo Deng, Chaoyou Xue, Xun Sun, Wei Wu, Sheng Bi, Nadeem Zubair, Guangchen Nie, Hui Xu

**Affiliations:** ^1^ Basic Medical College, Jiamusi University, Jiamusi, Heilongjiang, China; ^2^ Clinical Medical College, Jiamusi University, Jiamusi, Heilongjiang, China; ^3^ Public Health College, Jiamusi University, Jiamusi, Heilongjiang, China; ^4^ School of Mechanical Engineering, Jiamusi University, Jiamusi, Heilongjiang,, China; ^5^ Department of Orthopedics, The Fifth Hospital of Harbin, Harbin, Heilongjiang, China

**Keywords:** osteoarthritis, macrophage polarization, inflammation, bibliometric analysis, VOSviewer, immunopathology

## Abstract

**Background:**

Osteoarthritis, a common degenerative osteochondral disease, has a close relationship between its mechanism of Macrophage polarization. However, there are relatively few relevant studies in this field, and a more mature research system has not yet been formed.

**Methods:**

A bibliometric analysis was conducted using the Scopus and WoSCC databases to retrieve articles related to macrophages in OA published from 2013 to 2023. A total of 2,122 articles were analyzed for publication year, contributing countries, institutions, authors, journals, and keywords. VOSviewer software was used for co-authorship, co-citation, co-occurrence, and network visualization. Emerging research subtopics were also identified and reviewed.

**Results:**

The annual publication output showed a consistent upward trend. China led in the number of publications (623), with China Medical University contributing the most at the institutional level (41 articles). In contrast, the USA had the highest citation count (24,692), and Rush University Medical Center was the most cited institution (902 citations). *Frontiers Immunology* published the most articles (110), while *Osteoarthritis and Cartilage* received the highest number of citations (4,995). Chih-Hsin Tang was the most prolific author (16 publications), and Christin M. Lepus was the most frequently co-cited (2,085 citations). The most frequently occurring keywords included “osteoarthritis,” “metabolism,” “macrophage,” and “inflammation.” Researchers formed tightly connected teams with overlapping research themes.

**Conclusion:**

This study provides a comprehensive overview of global research on macrophages in OA, highlighting key contributors, journals, and emerging trends. Keyword cluster analysis identified future research directions, including metabolic reprogramming, macrophage polarization, and immune-modulation strategies. Greater standardization in research frameworks and enhanced international collaboration are needed to improve translational impact.

## 1 Introduction

Osteoarthritis (OA) is a degenerative joint disease caused by the destruction of articular cartilage integrity and lesions of the subchondral bone at the joint margins. Its symptoms include pain, swelling, stiffness, and restricted movement of the affected joints. OA has diverse causes, is difficult to diagnose in its early stages, and its pathogenesis remains unclear. It typically begins between the ages of 40 and 50, and by the age of 80, it affects nearly everyone to some extent. Among its various forms, knee osteoarthritis (KOA) is the most common (1, 2). OA is not merely a result of physical joint injury; long-term overload and biomechanical stress contribute to cartilage damage. This is often followed by inflammatory cell infiltration and various intracellular metabolic disorders, which further worsen the condition (3, 4) Currently, there is no cure for OA. Mainstream conservative treatments include oral non-steroidal anti-inflammatory drugs (NSAIDs) for pain relief and intra-articular injections, which can alleviate symptoms but have minimal effect in halting disease progression. These treatments also carry risks of serious side effects and complications such as infection.

Chondrocytes are the only cell type in articular cartilage and are essential for maintaining extracellular matrix (ECM) homeostasis. In osteoarthritis (OA), these cells undergo pathological changes, shifting toward a catabolic phenotype that overproduces inflammatory cytokines (e.g., IL-1β, TNF-α) and matrix-degrading enzymes (e.g., MMPs, ADAMTS), accelerating cartilage breakdown (5–7). This inflammatory milieu activates synovial macrophages, further amplifying joint damage (8).

Macrophages polarize into M1 (pro-inflammatory) and M2 (anti-inflammatory) subtypes, each playing opposing roles in OA progression (9). In recent years, macrophage polarization has emerged as a critical area of focus in osteoarthritis research due to its dual roles in mediating both pro-inflammatory and anti-inflammatory responses. The imbalance between these phenotypes contributes to persistent low-grade inflammation, synovial membrane remodeling, and cartilage breakdown hallmarks of OA pathophysiology (10). Consequently, targeting macrophage polarization offers promising therapeutic avenues for modulating disease progression (11, 12). Recent findings highlighted the bidirectional communication between macrophages and chondrocytes where each influences the other’s function and phenotype (13). However, the mechanistic details of this crosstalk remain poorly defined, and how it could be therapeutically targeted to restore joint homeostasis in OA remains an open question (14). Emerging evidence suggests that synovial inflammation, particularly the infiltration of macrophages and lymphocytes, plays a key role in OA pathogenesis (15). Recent study has been revealed the association between synovial inflammation and the development and onset of OA (16). Krasnokutsk et al. reported that patients with advanced OA exhibited a significantly higher prevalence of infrapatellar synovitis compared to those with early-stage disease (17, 18).

Synovial macrophages are highly abundant in OA joints, particularly within both the intimal and subintimal layers of the synovium (19). Sebastian et al. found that macrophages exhibited the most profound transcriptional changes in OA progression following joint injury (20). These findings suggest that macrophages not only contribute to disease initiation but may also serve as therapeutic targets in OA. Despite a growing body of literature, there is a lack of systematic evaluations mapping the developmental trends, key contributors, and emerging research hotspots in this field. Bibliometric analysis offers a quantitative method to explore scientific output and identify evolving trends, influential authors, and collaborative networks using tools like VOSviewer (21, 22).

This study aimed to conduct a comprehensive bibliometric analysis of global publications on macrophages in OA from 2013 to 2023 analyzing publication trends, identifying leading countries, institutions, journals, and authors, exploring keyword clusters, examining the evolution of research focus and methodological approaches, evaluating efforts to target polarized macrophages in OA, and highlighting opportunities for international collaboration and translational advancement.

## 2 Materials and methods

### 2.1 Data source and search strategies

Based on a review of previous bibliometric studies, the current analysis was conducted using two major scientific databases: Scopus (www.scopus.com) and the Science Citation Index Expanded (SCIE), a sub-database of the Web of Science Core Collection (WoSCC) (www.webofscience.com) (23, 24).

The publication time frame was set from 2013 to 2023, and only documents published in English were included. Document types were restricted to original articles and review papers. The topic of interest was Global Research Trends on Macrophage Polarization in Osteoarthritis: A Bibliometric and Visual Analysis (2013–2023). The search strategy followed a structured process **Table 1**.

**Table 1:** Detailed steps of the literature search strategy.

1	Identification of Key Concepts	Macrophages and Osteoarthritis.
2	Determination of Synonyms and Related Terms	Macrophages: immune cells, monocytes, macrophage polarization.
		Osteoarthritis: OA, degenerative joint disease, osteoarthrosis.
3	Construction of Boolean Search Query.	The final query used was: (macrophages OR “immune cells” OR monocytes OR “macrophage polarization”) AND (Osteoarthritis OR OA OR “degenerative joint disease” OR osteoarthrosis)

**Figure 1:**
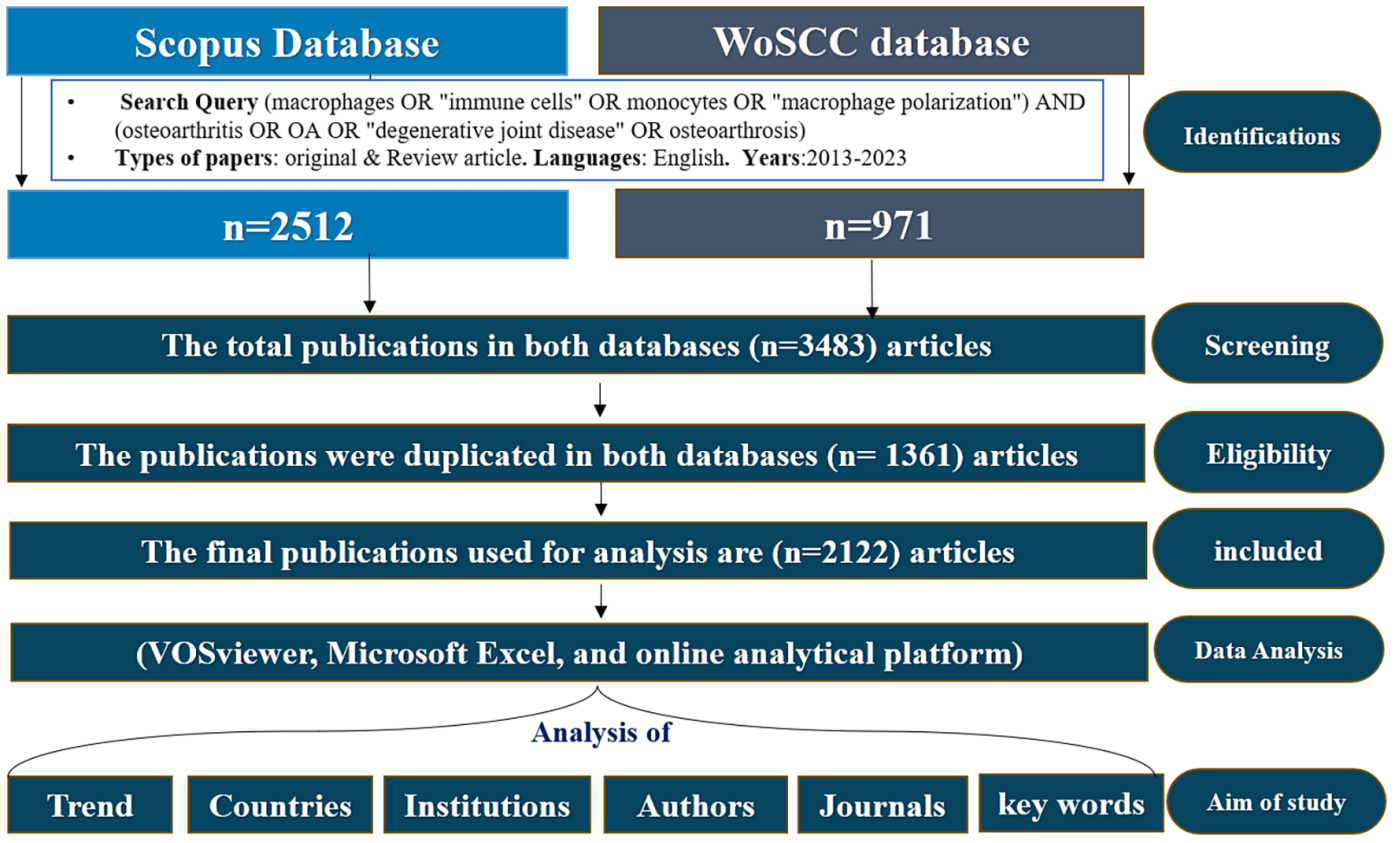
Data processing flow chart of bibliometric analysis.

Researchers independently executed the search approach twice and then cross-verified it to mitigate search bias and omissions. And search was conducted in both databases, and the selection process followed the PRISMA (Preferred Reporting Items for Systematic Reviews and Meta-Analyses) guidelines (25). The complete literature selection process is presented in **Figure 1** Since this analysis was based solely on secondary data from published articles, ethical approval was not required.

### 2.2 Inclusion and exclusion criteria

All documents used for bibliometric analysis met the following criteria: (1) The documents were published between 2013 and 2023. (2) The language of publication was English. (3) The article types are original articles and review articles. Papers that do not meet these criteria are excluded, this criteria is are based previous bibliometric studies (23, 24).

### 2.3 Data collection and processing software

Terms were automatically derived from the titles, abstracts and keywords of all papers in the datasets. These were utilized to create maps like networking and density visualizations based on textual data (26). We obtained 3483 related papers, which were reviewed based on the inclusion and exclusion criteria and finally 2122 papers were used in the analysis after removed duplications data using duplication detection tools as well as looking at the title and abstract. These documents were saved as full records, citation references plain text to generate the source files for analysis using Microsoft Excel 2019 (Microsoft Corporation, Redmond, WA, USA) and SPSS version 21.0 (IBM Corp., Armonk, NY, USA). Bibliometric analysis Bibliometric analysis VOSviewer (version 1.6.16) (27) were assessed, including annual trends in publications and citations, contributing countries, contributing institutions, contributions of journals and cited journals, prolific authors and Cited Authors, keyword frequency, cluster, and research fields. Additionally, journal impact factors (IF) and quartile rankings (Q1–Q4) were obtained from the Journal Citation Reports (JCR). The search was carried out on July/15/2024 as shown in **Figure 1**.

### 2.4 Bibliometric and visualization analysis

Bibliometric analysis was performed using VOSviewer (version 1.6.16) for co-authorship analysis, journal co-citation networks, keyword co-occurrence, and mapping institutional collaboration networks. Three types of visualizations were generated: Network, Overlay, and Density Visualization. Node size represents the frequency of occurrence publications or citations, while link thickness shows the strength of relationships. The Total Link Strength metric quantified the overall strength of these associations. The trends identified through VOSviewer, including macrophage polarization, align with findings from studies using similar bibliometric methods. These studies also employed VOSviewer and highlighted the growing importance of macrophage-related research, confirming the robustness of our findings (28–30). VOSviewer’s network clarity enabled the clear detection of clusters and research directions, supporting the interpretation of complex relationships and reinforcing the conclusions of this study. The results are consistent with tool-independent observations, ensuring the reliability of the identified trends in OA macrophage research (27).

### 2.5 Statistical analysis

Descriptive statistical analyses were performed using SPSS version 21.0 (IBM Corp. Armonk, NY, USA) and Microsoft Excel 2019. Categorical variables were presented as frequencies & percentages.

## 3 Results

### 3.1 Analysis of publication trends

**Figure 2:**
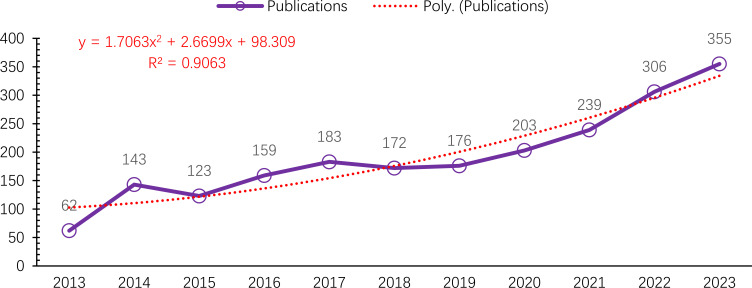
Visual graph of publication trend.

A total of 2,122 publications met the inclusion criteria and were analyzed bibliometrically **Figure 1**. From 2013 to 2023, the annual number of publications on macrophages associated with OA showed a steady upward trend, despite minor fluctuations in 2015 and 2018. The number of publications increased markedly from 62 in 2013 to 355 in 2023 **Figure 2**. This growth followed a quadratic trendline described by the equation y = 1.7063x² + 2.6699x + 98.309, with a strong coefficient of determination (R² = 0.9063), indicating a consistent rise in research activity. The temporary decline in 2015 (123 publications) and the moderate output in 2018 (172 publications) were followed by a significant increase from 2019 onwards. In 2019, 176 articles were published, with a steady year-on-year rise that peaked at 355 publications in 2023. This upward trajectory underscores the growing scientific interest in the immunological and pathological roles of macrophages in osteoarthritis pathogenesis (13, 31).

### 3.2 Analysis of the counties

**Figure 3:**
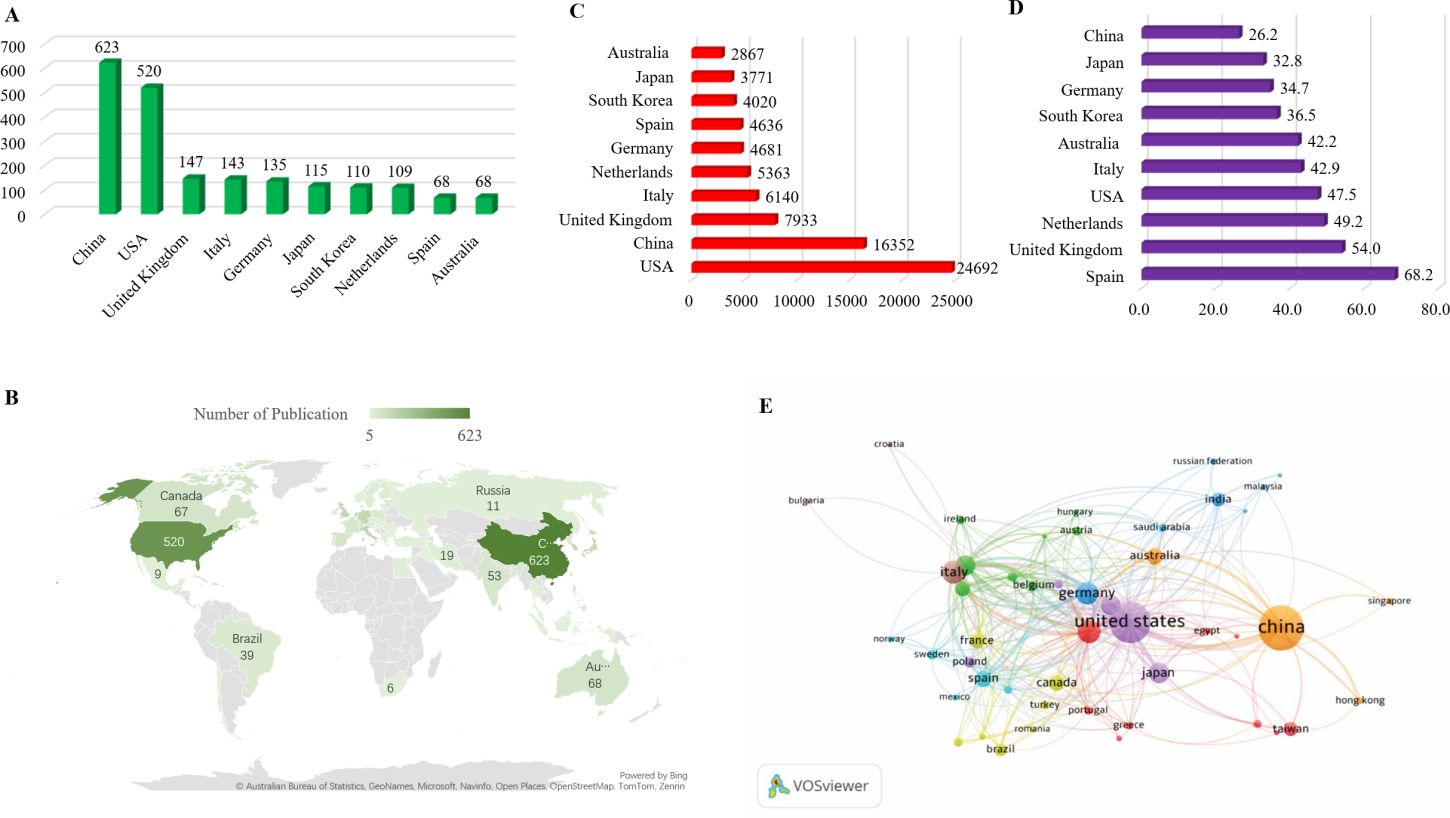
**(A)** List the top 10 countries contributing to publications. **(B)** Distribution of Macrophages associated with osteoarthritis in World Map among 48 countries have more than 5 publications. **(C)** Total number of citations of top 10 countries. **(D)** average number of citations of top 10 countries. **(E)** Visualization of co-authorship and publication volume by country using VOSviewer.

From 2013 to 2023, research on macrophages associated with osteoarthritis was published in 98 countries. To better understand the global distribution and collaborative landscape, the Co-authorship-Countries function in VOSviewer was applied, with a maximum of 25 countries per article. This analysis identified 48 countries that published more than five papers on the topic, with their distribution visualized. China led the field in terms of publication output, contributing 623 articles, followed by the United States (520 publications) and the United Kingdom (147 publications). Together, these three countries accounted for 39.4% of the total global publications in this area highlighting their dominant role in advancing research on macrophages in osteoarthritis. A second tier of active contributors included Italy (143 publications), Germany (135), Japan (115), South Korea (110), and the Netherlands (109). Spain and Australia also showed substantial engagement in this field **Figure 3A**.

To illustrate international collaboration, a co-authorship map was generated using VOSviewer, depicting the interactions among the 48 countries with more than five relevant publications during the study period **Figure 3B**. This map reveals dense connections and cross-national cooperation, particularly among leading countries. In terms of citation trends, the United States ranked first with 24,692 total citations, followed by China (16,352) and the United Kingdom (7,933). Other countries including Italy, the Netherlands, Germany, Spain, South Korea, Japan, and Australia also received considerable citation attention for their work in this area **Figure 3C**. Regarding average citation impact, Spain led with an average of 68.2 citations per publication, followed by the United Kingdom (54.0) and the Netherlands (49.2). The USA, Italy, Australia, South Korea, Germany, Japan, and China had average citation counts ranging from 47.5 to 26.2, with a notable decline after the top three **Figure 3D**. Visualization characteristics are further detailed in **Figure 3E**. In this map, node size indicates the number of publications (larger nodes represent higher output), while node color represents collaboration intensity. Brighter shades denote stronger collaborative links, highlighting the active participation and interconnectedness of the leading countries in macrophage-related osteoarthritis research.

### 3.3 Analysis of the institutions

**Figure 4:**
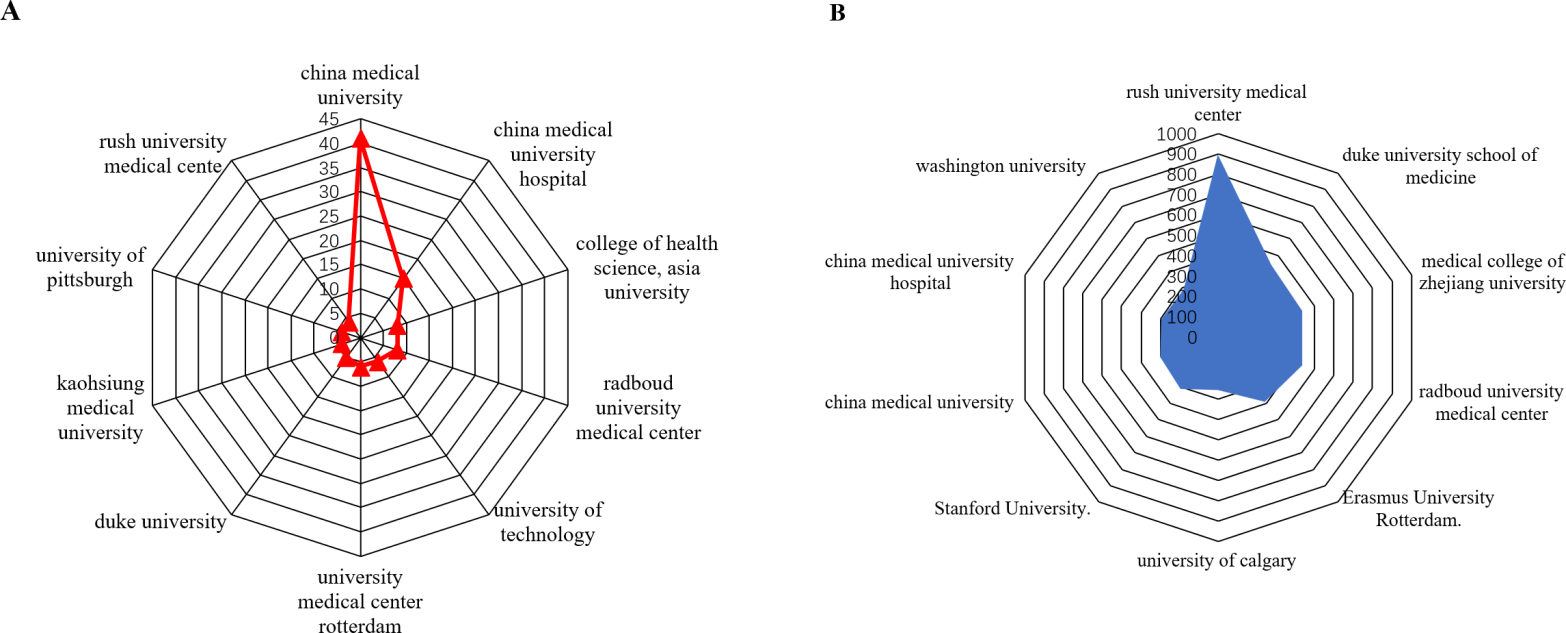
**(A)** is analysis of the top 10 institutions with the highest number of published studies. **(B)** analysis of the top 10 institution’s citation.

As shown in **Table 2** The top 10 institutions contributing to publications **Table 2** the top 10 most productive institutions in the field of macrophage-related osteoarthritis research were identified among a total of 7,284 articles, with 92 institutions contributing more than three publications each. China Medical University ranked first with 41 publications, followed by China Medical University Hospital (15), College of Health Science, Asia University (8), Radboud University Medical Center (8), University of Technology, Netherlands (6), University Medical Center Rotterdam (6), Duke University (5), Kaohsiung Medical University (4), University of Pittsburgh (4), and Rush University Medical Center (4), as shown in **Figure 4A**. These institutions have made notable academic and scientific contributions. However, the analysis revealed limited inter-institutional collaboration, indicating gaps in connectivity within the global research network.

**Table 2:** The top 10 institutions contributing to publications.

Rank	Institutions	Publications	Affiliation
1	China medical university	41	China
2	China Medical University Hospital	15	China
3	college of health science, Asia university	8	Taiwan
4	Radboud University Medical Center	8	Netherlands
5	university of technology	6	Netherlands
6	University Medical Center Rotterdam	6	United States
7	duke university	5	Taiwan
8	Kaohsiung medical university	4	United States
9	university of Pittsburgh	4	United States
10	Rush University Medical Center	4	United States

As presented in **Table 3**, Rush University Medical Center (Chicago, IL, USA) led in total citations with 902, followed by Duke University School of Medicine (445), Medical College of Zhejiang University (434), Radboud University Medical Center (434), Erasmus University Rotterdam (389), Stanford University (314), University of Calgary (260), China Medical University (302), China Medical University Hospital (297), and Washington University (291). As illustrated in **Figure 4B**. citation performance highlights the significant academic impact of these institutions in advancing the understanding of macrophage roles in osteoarthritis. Notably, recent studies from Rush University Medical Center have emphasized the critical role of synovial macrophages in osteoarthritis progression. These findings underscore the therapeutic potential of targeting macrophage activity to manage or possibly reverse disease progression (32).

**Table 3:** The top 10 most cited institutions.

Rank	Institutions	Citation	Affiliation
1	Rush University Medical Center	902	United States
2	Duke university school of medicine	445	United States
3	Medical College of Zhejiang University	434	China
4	Radboud University Medical Center	434	Netherlands
5	Erasmus University Rotterdam.	389	Netherlands
6	University of Calgary	260	Canada
7	Stanford University.	314	United States
8	China medical university	302	China
9	China Medical University Hospital	297	China
10	Washington University	291	United States

### 3.4 Analysis of journals and cited journals

**Figure 5:**
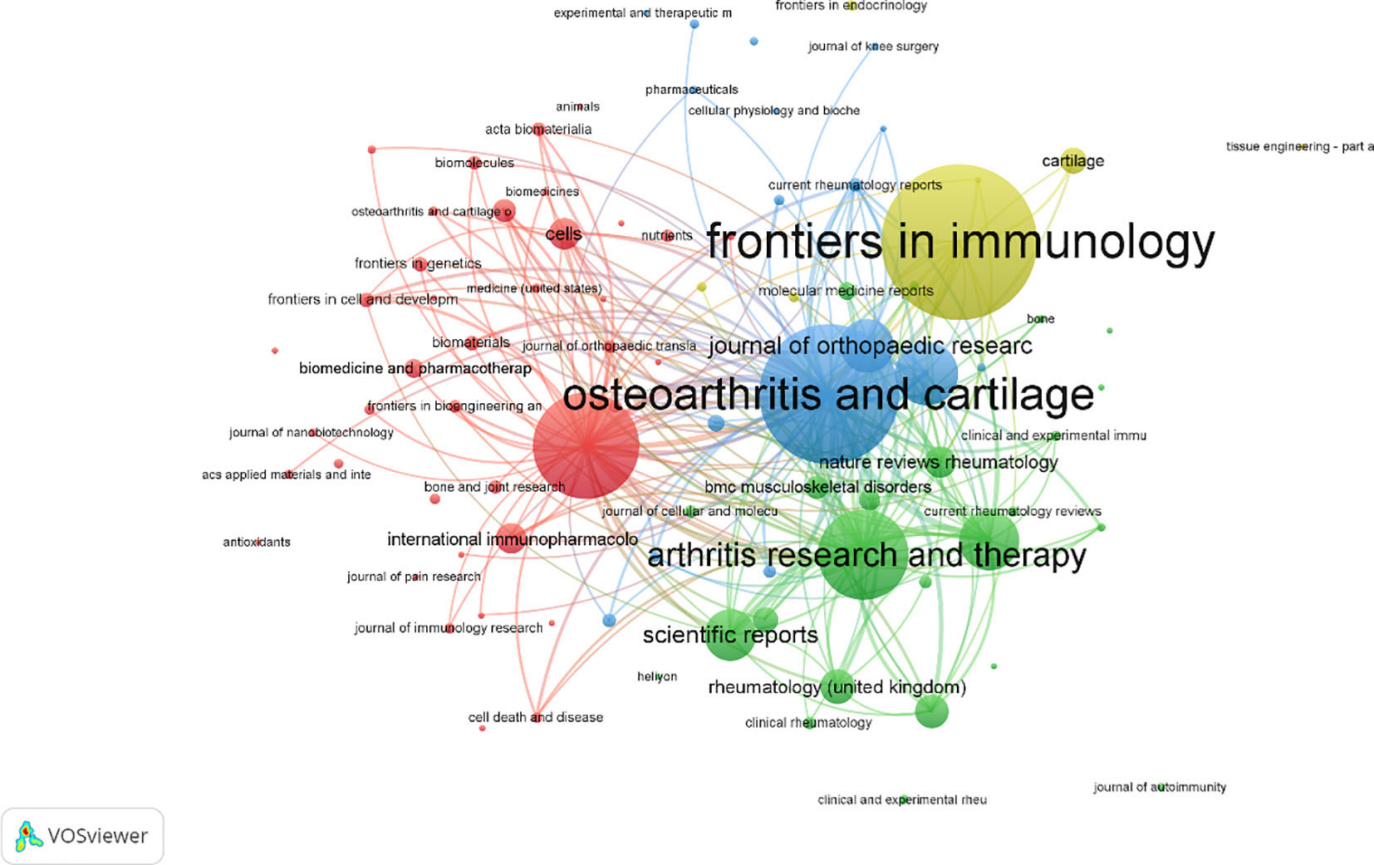
Visualization of journal article volume in macrophages associated with osteoarthritis.

A total of 558 publications were identified across various journals in the field of macrophage-related osteoarthritis research. The top 10 journals accounted for 26.3% of the total output, underscoring their central role in disseminating findings in this domain. *Frontiers in Immunology* published the highest number of articles, and the average impact factor (IF) of these core journals reached 6.87, with all falling within the Q1 quartile, reflecting the high quality and influence of research published in this area. Among these, the *Annals of the Rheumatic Diseases* had the highest impact factor (IF = 20.3), as presented in **Table 4** the journal co-citation network. **Figure 5** comprised 681 nodes and 2,730 linkages, representing the interrelationships among journals. In this network, nodes represent individual journals, lines indicate co-citation relationships, colors reflect publication years, and node size corresponds to citation frequency.

**Table 4:** The top 10 journals contributing publications.

Rank	Journal	Impact factor	JCR quartile	Publications
1	Frontiers in Immunology	5.9	Q1	110
2	Osteoarthritis and Cartilage	7.2	Q1	99
3	International Journal of Molecular Sciences	4.9	Q1	75
4	Arthritis Research and Therapy	4.4	Q1	64
5	Plos One	2.9	Q1	45
6	Scientific Reports	3.8	Q1	37
7	Journal of Orthopedic Research	2.8	Q1	38
8	Arthritis and Rheumatology	10.9	Q1	43
9	Annals of the Rheumatic Diseases	20.3	Q1	24
10	Cells	6.0	Q1	23

**Figure 6:**
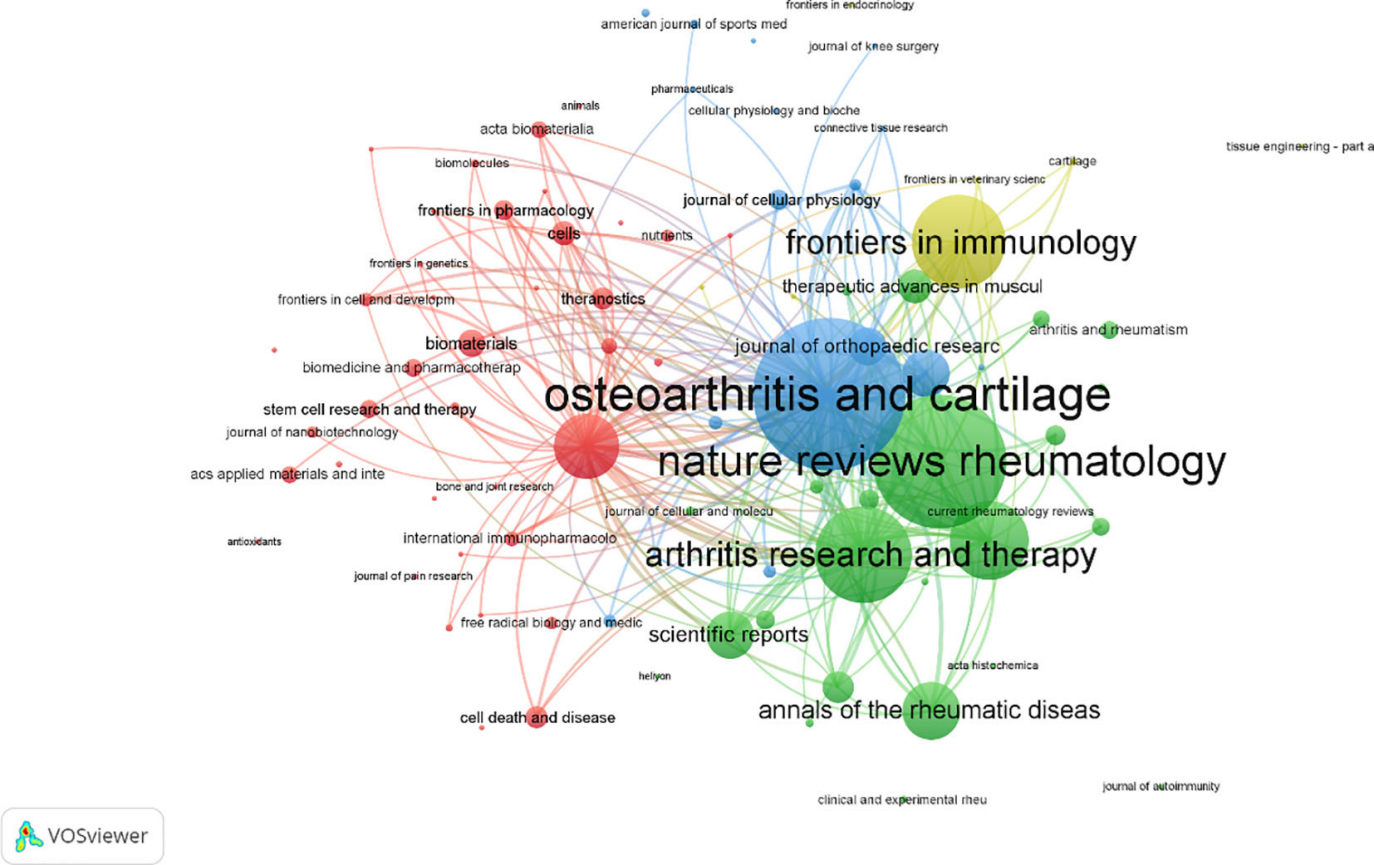
Visualization of cited journal in macrophages associated with osteoarthritis.

As shown in **Table 5**, the top 10 journals were ranked based on total link strength and citation frequency. *Osteoarthritis and Cartilage* ranked first, with 4,995 citations and a total link strength of 257, highlighting its authoritative status in the field. This finding is further supported by the co-citation network visualization in **Figure 6**. Notably, the most frequently cited article in *Osteoarthritis and Cartilage* discussed the senolytic agent Quercetin, which was shown to mitigate intervertebral disc degeneration via the Nrf2/NF-κB signaling axis. This highlights the importance of the NLRP3 inflammasome and NF-κB pathways in linking osteoarthritis to intervertebral disc pathology. The findings suggest that Quercetin may modulate macrophage-mediated inflammation by influencing these signaling pathways (33) Additionally, journals belonging to the field of Endocrinology & Metabolism such as *Osteoporosis International*, *Journal of Bone and Mineral Research*, and Bone were the leading journals involved in the publication of research on macrophage and OA (34).

**Table 5:** The top 10 most cited journals.

Rank	Journal	Impact factor	JCR quartile	Citations	Total link strength
1	Osteoarthritis and Cartilage	7.2	Q1	4995	257
2	Nature Reviews Rheumatology	32.2	Q1	4293	36
3	Arthritis Research and Therapy	4.4	Q1	3032	75
4	Frontiers in Immunology	5.9	Q1	2916	16
5	Arthritis and Rheumatology	10.9	Q1	2402	86
6	International Journal of Molecular Sciences	4.9	Q1	1960	92
7	Annals of the Rheumatic Diseases	20.3	Q1	1704	78
8	Scientific Reports	3.8	Q1	1376	24
9	Plos One	2.9	Q1	1359	16
10	Journal of Orthopedic Research	2.8	Q1	1063	47

### 3.5 Analysis of the authors

**Figure 7:**
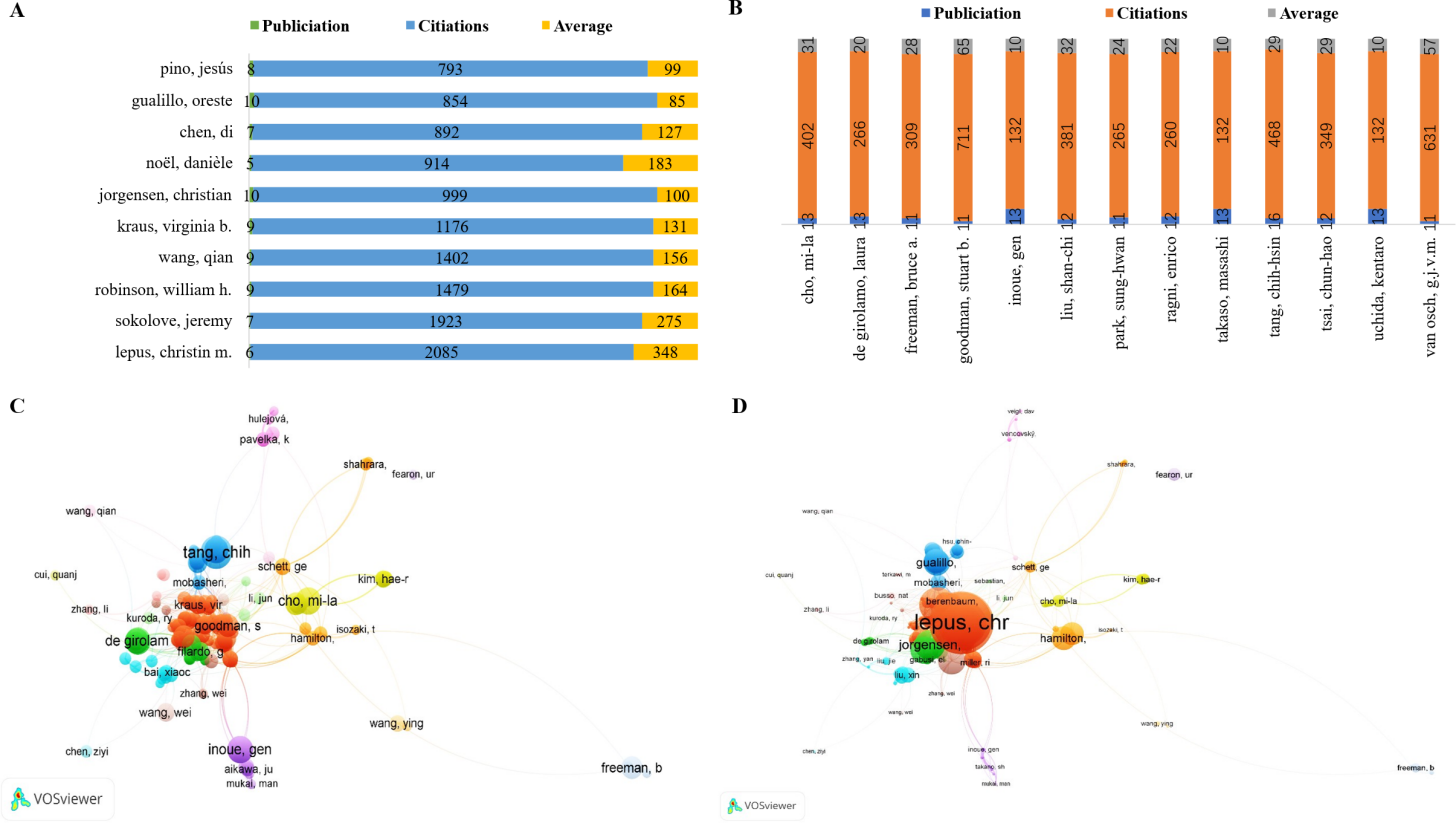
**(A)** The top 10 authors with the most publications. **(B)** The top 10 authors with the most citation accounts. **(C)** Visualization of author volume in macrophages associated with osteoarthritis. **(D)** Visualization of cited author in macrophages associated with osteoarthritis.

The analysis identified the top 10 most productive authors in macrophage-related osteoarthritis research. Chih-Hsin Tang led with 16 publications, followed closely by Kentaro Uchida, Masashi Takaso, Laura De Girolamo, Gen Inoue, and Mi-La Cho, each with 13 publications. Chun-Hao Tsai and Enrico Ragni followed with 12 publications each, while Shan-Chi Liu also made notable contributions with 12 articles. Additionally, Stuart B. Goodman, G.J.V.M. van Osch, Bruce A. Freeman, and Sung-Hwan Park each had 11 publications **Figure 7A**. Among these prolific authors, Chih-Hsin Tang’s studies have notably addressed the relationship between OA, aging, and obesity. One key study demonstrated that omentin-1, a protective adipokine, is reduced in OA patients and plays an anti-inflammatory role by promoting IL-4-dependent M2 macrophage polarization in synovial fibroblasts. This regulation occurs through the PI3K, ERK, and AMPK pathways, ultimately suppressing inflammatory mediators, preventing cartilage degradation, and limiting bone erosion in OA models (35). The second most prolific author, Mi-La Cho from The Catholic University of Korea (Medical College), has extensively explored immunological signaling pathways and inflammatory mechanisms in arthritis. Her research includes the impact of metformin, which attenuates experimental autoimmune arthritis by balancing Th17/Treg cells and inhibiting osteoclastogenesis mechanisms likely relevant to macrophage polarization and metabolic reprogramming in OA (36), Furthermore, Cho has investigated STA-21, a potential STAT3 inhibitor, and its anti-arthritic effects in IL-1Ra knockout rats, providing insights into new treatment strategies for inflammatory arthritis (37). The most cited authors in this field are led by Christin M. Lepus with 2,085 citations, followed by Jeremy Sokolove (1,923), William Robinson (1,479), Qian Wang (1,402), Virginia B. Kraus (1,176), Christian Jorgensen (999), Danièle Noël (914), Di Chen (892), Oreste Gualillo (854), and Jesús Pino (793), as shown in **Figure 7B**. Christin M. Lepus, from the Department of Immunology and Rheumatology at Stanford University School of Medicine, has demonstrated that OA involves cartilage degradation, bone remodeling, and synovial inflammation, with macrophages playing a pivotal role in sustaining low-grade chronic inflammation (38). Her work has emphasized that macrophage polarization presents a promising target for disease-modifying treatments, particularly as current therapies like NSAIDs and glucocorticoids only offer symptom relief and may accelerate cartilage damage with prolonged use (39, 40). In total, 152 authors were identified as having published more than five articles, and 2,536 cooperative links were visualized among them. These collaborations, along with bibliographic coupling (based on publication frequency) and co-citation networks (based on citation frequency), provide insight into leading contributors and research trends over time. In the visual network **Figures 7C, D**, node size represents the number of publications or citations, line thickness reflects the strength of collaborations or co-citations, and node color corresponds to publication year, illustrating the temporal evolution of author influence.

### 3.6 Analysis of keywords

A keyword co-occurrence analysis was conducted to identify research hotspots and evolving trends in the field of macrophages associated with OA. In this analysis, a co-occurrence link is formed when two keywords appear together in the same publication, indicating thematic connections. Keyword clustering reveals how topics are interrelated by evaluating the frequency and strength of these co-occurrences. Out of 17,866 total keywords, 2,838 keywords with five or more occurrences were selected for visualization. The resulting co-occurrence network is depicted in **Figure 8A**, highlighting the density and connectivity of terms within the research landscape.

**AFigure 8:**
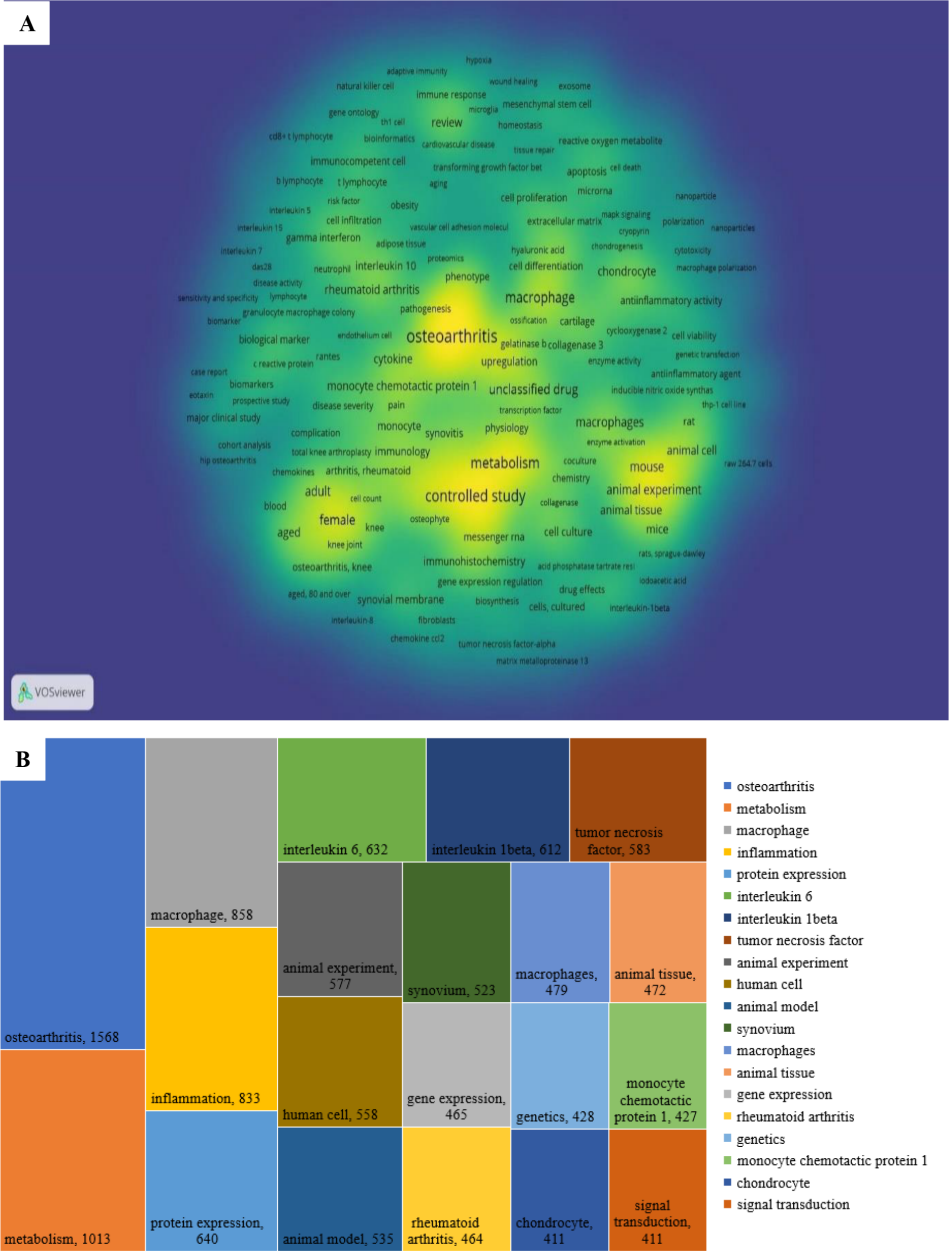
**(A)** Network density of keywords. **(B)** The 20 most frequently occurring keyword.

**Figure 8B** presents the top 20 most frequently occurring keywords. The most prevalent was osteoarthritis (n = 1568), followed by metabolism (n = 1013), macrophage (n = 858), inflammation (n = 833), protein expression (n = 640), interleukin-6 (n = 632), interleukin-1β (n = 612), tumor necrosis factor (n = 583), human cell (n = 558), animal model (n = 535), synovium (n = 523), animal tissue (n = 472), animal experiment (n = 472), gene expression (n = 465), rheumatoid arthritis (n = 464), genetics (n = 428), monocyte chemotactic protein-1 (n = 427), chondrocyte (n = 411), and signal transduction (n = 411).

Among these, metabolism plays a key role in the reprogramming of macrophages, which is essential for their immune functions. M1 macrophages rely predominantly on glycolysis to produce pro-inflammatory cytokines such as TNF-α and IL-6, whereas M2 macrophages utilize oxidative phosphorylation and fatty acid oxidation, enabling anti-inflammatory responses and tissue repair. This metabolic flexibility allows macrophages to adapt to the inflammatory or reparative needs in OA (31).

Gene expression also contributes significantly to macrophage polarization. M1 macrophages upregulate genes encoding pro-inflammatory cytokines such as TNF-α and IL-1β, whereas M2 macrophages enhance anti-inflammatory genes like IL-10 and TGF-β (11). These processes are regulated by transcription factors including NF-κB, STAT1, STAT6, and PPARγ (41). The mechanism of growth factors in healing significantly influences macrophage polarization and the course of OA. through binding to certain cell surface receptors (11). Growth factors further modulate macrophage polarization by activating signaling pathways like MAPK and PI3K/AKT, which influence gene expression and protein synthesis, ultimately determining M1 or M2 phenotypes (42, 43). Cytokines such as IL-6 and IL-1β emerged as prominent keywords due to their well-documented roles in OA pathogenesis. IL-6 levels are elevated in both the synovial fluid and serum of OA patients, correlating with disease severity and contributing to cartilage breakdown (44). IL-1β and tumor necrosis factor (TNF) are also central to inflammation and cartilage degradation (45). Monocyte chemotactic protein-1 (MCP-1) is another notable keyword, signifying its importance in recruiting monocytes/macrophages to inflamed joints. MCP-1 exacerbates inflammation and cartilage damage, positioning it as a potential target for modulating immune infiltration in OA (46). Chondrocytes, the primary cells responsible for maintaining cartilage integrity, also featured prominently. In OA, dysfunctional chondrocytes contribute to matrix degradation. The interaction between chondrocytes and inflammatory cytokines such as IL-6, IL-1β, and TNF is crucial for developing strategies aimed at cartilage preservation and repair through the modulation of macrophage activity (47).

Emerging evidence underscores the growing significance of exosomes in regulating macrophage behavior in OA. Recent studies highlight the potential of exosome-based therapies for modulating macrophage polarization and promoting tissue repair in OA. Nguyen et al. demonstrated that exosomes containing growth factors and siRNAs can significantly influence macrophage proliferation, migration, and polarization in a dose-dependent effect (48). A recent study by Wang H et al. showed that exosomes derived from miR-146a-overexpressing fibroblast-like synoviocytes (FLS) modulate macrophage polarization from the pro-inflammatory M1 phenotype to the anti-inflammatory M2 phenotype. This shift occurs via the TLR4/TRAF6/NF-κB signaling pathway, which leads to reduced synovial inflammation and cartilage degradation in OA rats. This suggests that miR-146a-enriched FLS-derived exosomes could serve as novel therapeutic agents for OA, influencing macrophage behavior and promoting cartilage repair (49). Furthermore, a systematic review analyzed the therapeutic potential of exosomes derived from various stem cells, particularly mesenchymal stem cells (MSCs), for OA treatment. The review highlighted how MSC-derived exosomes can modulate macrophage polarization, reduce inflammation, and promote cartilage regeneration. These findings further reinforce the growing interest in exosome-based therapies as a promising strategy for OA management (50). Finally, the keyword *in vitro* showed the highest total link strength, underscoring its pivotal role in bridging basic science and clinical translation. Despite challenges in clinical trials due to variable methodologies and patient heterogeneity, *in vitro* studies remain critical for elucidating the mechanisms and therapeutic potential of macrophages in OA.

### 3.7 Analysis of clusters keywords

**Figure 9:**
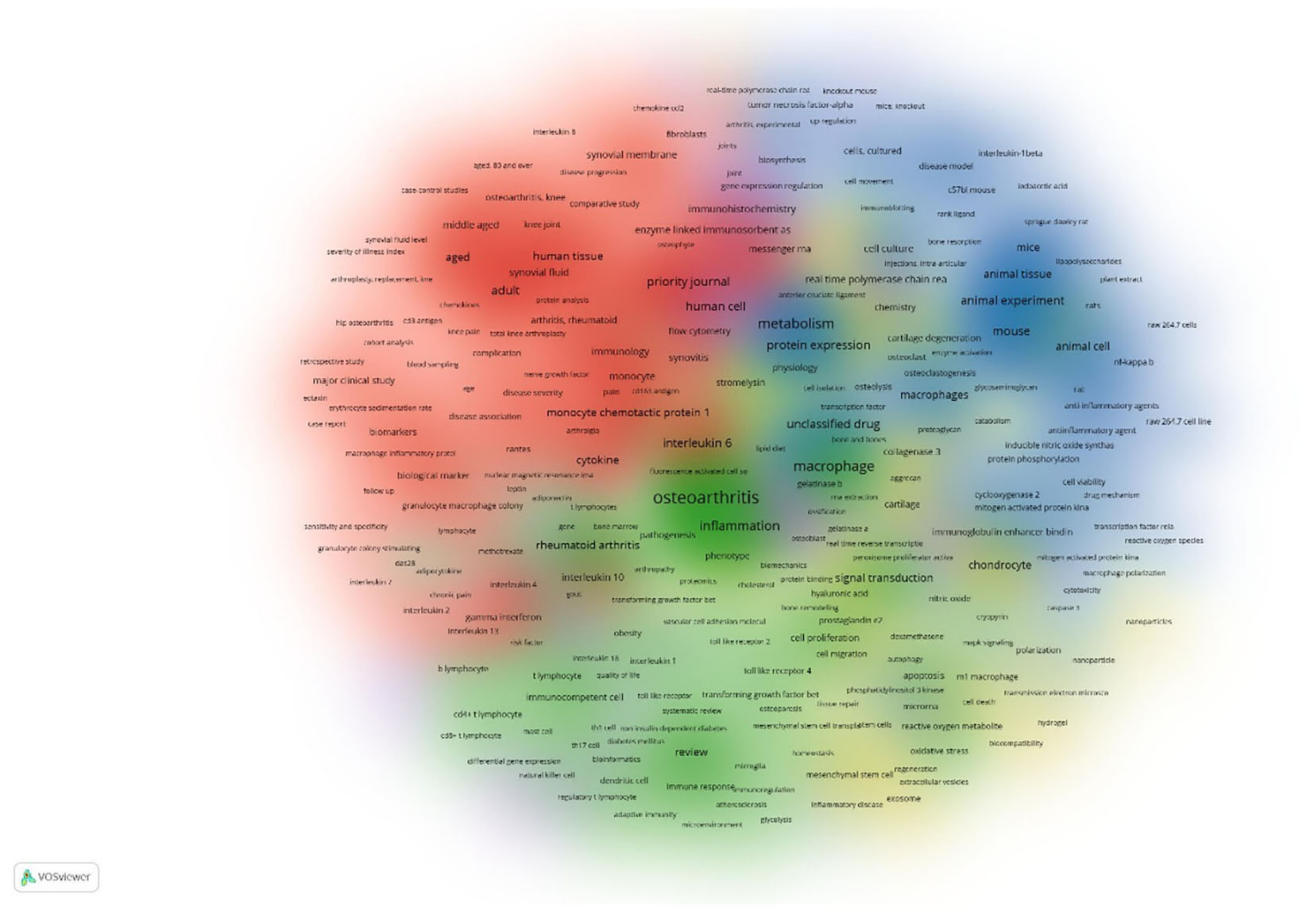
Cluster density Classification on the co-occurrence on the authors’ keywords the colors in the nodes represent different types of clusters, while the size of each node indicates the frequency of the keywords. appearance in the research. Larger nodes show more frequently occurring keywords.

A keyword co-occurrence cluster analysis was performed with a threshold of ≥5 occurrences per keyword, resulting in 510 keywords categorized into six distinct clusters, each represented by a unique color in the visual network map **Figure 9**. These clusters reveal research subfields and strategic directions in the study of macrophages associated with osteoarthritis (OA):

#### 3.7.1 Cluster 1 – biomarkers and clinical indicators in OA

This cluster centers on biomarkers, inflammatory cytokines, immune cells, and clinical indicators, particularly in knee osteoarthritis. It highlights the role of immunological mediators (e.g., interleukins, chemokines) and diagnostic tools such as MRI and radiography in assessing disease progression, pain severity, and therapeutic efficacy.

#### 3.7.2 Cluster 2 – immunopathology and autoimmune inflammation

Focused on the immunological responses in chronic inflammatory diseases, especially rheumatoid arthritis, this cluster explores both innate and adaptive immunity. Key terms include T lymphocytes, cytokines, and processes like autophagy and inflammation, as well as therapeutic strategies involving immunomodulatory agents and disease-modifying antirheumatic drugs (DMARDs).

#### 3.7.3 Cluster 3 – experimental models and molecular mechanisms

This cluster emphasizes *in vitro* and *in vivo* studies utilizing models such as C57BL/6 mice and Sprague-Dawley rats to investigate inflammatory pathways, drug effects, and macrophage polarization (M1/M2). It includes MAPK, NF-κB, and other signaling cascades and techniques like Western blotting and immunofluorescence, reflecting a focus on anti-inflammatory therapies and molecular drug targets.

#### 3.7.4 Cluster 4 – cartilage repair and regenerative medicine

Keywords in this cluster relate to mesenchymal stem cells, chondrocyte proliferation, and extracellular matrix components (e.g., collagen, proteoglycans), crucial for tissue engineering and cartilage regeneration. This group also features enzymes such as matrix metalloproteinases (MMPs) and aggrecanases, highlighting cartilage degradation and repair processes central to OA pathology.

#### 3.7.5 Cluster 5 – gene expression and bioinformatics approaches

This cluster involves techniques for molecular analysis including RNA-seq, RT-qPCR, and gene expression profiling. It focuses on transcription factors, protein interactions, and bioinformatics tools like flow cytometry and microarrays, which are essential for investigating macrophage gene regulation, immune signaling, and cellular communication in the OA microenvironment.

#### 3.7.6 Cluster 6 – bone remodeling and osteoimmunology

Centered on bone biology, this cluster explores bone metabolism, osteoblast/osteoclast activity, and transcription factors like RUNX2. Key molecules such as RANKL, osteoprotegerin, and methodologies like micro-CT and histology are prominent, reflecting research into subchondral bone changes, bone remodeling, and their links to OA progression.

These six clusters provide an in-depth representation of current research themes and methodological approaches, offering valuable insight into both the molecular underpinnings and therapeutic possibilities in the field of OA-related macrophage biology.

## 4 Discussion

### 4.1 Evolution of research focus and methodological approaches

Our bibliometric analysis reveals a distinct shift in the scientific understanding of osteoarthritis (OA). Once considered a purely mechanical “wear-and-tear” condition, OA is now increasingly recognized as a multifactorial disease with significant inflammatory and immunological components (51). A key aspect of this paradigm shift is the growing attention to the role of macrophages in OA pathogenesis, as evidenced by the rising number of publications addressing this topic over the past two decades (39). In the early years (pre-2010), studies primarily established correlative links between macrophage infiltration in synovial tissues and OA progression, without clearly defining causal mechanisms (52). However, more recent investigations have transitioned toward mechanistic insights, exploring how different macrophage phenotypes particularly the M1 (pro-inflammatory) and M2 (anti-inflammatory) subtypes modulate disease initiation, progression, and tissue repair processes (53).

This conceptual evolution has been paralleled by significant methodological advancements. Early investigations largely depended on histopathology, immunostaining, and basic *in vitro* assays to assess cellular changes (53). In contrast, contemporary studies now leverage cutting-edge techniques such as single-cell RNA sequencing, spatial transcriptomics, and high-resolution *in vivo* imaging to dissect macrophage heterogeneity, plasticity, and functional behavior in OA-affected joints (54). These sophisticated approaches have uncovered distinct roles for tissue-resident versus infiltrating macrophages in maintaining joint homeostasis and mediating inflammatory responses—insights that were previously inaccessible. Together, these developments underscore a growing complexity in OA research, emphasizing the interplay between biomechanics, inflammation, and immune cell dynamics. They also reflect a more precise and integrated approach to studying macrophage function in OA, potentially paving the way for novel therapeutic strategies targeting specific immune pathways.

### 4.2 Geographical distribution and collaboration networks

The dispersion of the knowledge on the role of macrophage in OA across the world has shown some of the strong points, and the gaps that need to be addressed with critical requirements in scientific works. Although North America, Western Europe, and East Asia persistently dominate publication and research output and impact, those nations in Africa, South America and some areas of the Middle East regions with high OA burdens continue to be underrepresented (55). Such a geographic difference restricts the equity of research conducted worldwide and casts the question of whether the current findings can be generalized to the populations that are genetically, environmentally, and culturally diverse (56). This territorial imbalance only helps to restrict research equality worldwide, but it also creates significant concerns regarding the generalizability of the available research to populations which are genetically, environmentally, and culturally diverse (57). The influence of local factors, such as diet, physical activity, and comorbid conditions, may affect macrophage activity and OA pathogenesis. Without broader representation, therapeutic strategies from high-income countries may not fully address the needs of patients in underrepresented areas.

Our analysis of collaboration networks shows that high-impact research often arises from interdisciplinary teams combining immunology, rheumatology, orthopedics, biomedical engineering, and bioinformatics (58). These cross-disciplinary efforts enhance methodological robustness and conceptual innovation. However, we also observed that such collaborative clusters are frequently confined within specific countries or regions, limiting global knowledge integration (59). To address these gaps, fostering more extensive international collaborations particularly those involving low- and middle-income countries is essential. Such partnerships could enrich the research landscape with broader patient demographics, enhance scientific innovation through diverse perspectives, and ultimately lead to more globally relevant and effective therapeutic interventions for OA.

### 4.3 Interdisciplinary collaboration

Interdisciplinary research is becoming increasingly central to advancing our understanding of OA and its related to the macrophage polarization. Partnerships between immunology and biomedical engineering have been pivotal in developing biomaterial-based approaches to modulate macrophage behavior and promote tissue repair (58). Bioinformatics collaborations are also emerging as essential, particularly in analyzing large datasets to understand macrophage polarization patterns in OA joints. Studies using advanced bioinformatics tools to investigate gene expression and macrophage behavior offer new therapeutic insights (60, 61). Recent studies exemplifying these interdisciplinary efforts include the development of macrophage-targeted biomaterial scaffolds designed to promote tissue repair and modulate macrophage polarization in OA joints (62). Nanotechnology delivering anti-inflammatory agents to macrophages combines immunology, engineering, and materials science for precise OA treatments (63). Collaboration between rheumatology and bioinformatics has also led to breakthroughs in understanding how macrophages interact with chondrocytes and the extracellular matrix during disease progression, further bridging clinical and computational research (64). These collaborative efforts demonstrate how interdisciplinary partnerships are driving innovation in OA macrophage research. Expanding these collaborative networks to include a more diverse range of countries and expertise will enhance the development of innovative solutions for OA treatment and improve the overall global health impact of OA research.

### 4.4 Targeting macrophages polarization in osteoarthritis

**Figure 10:**
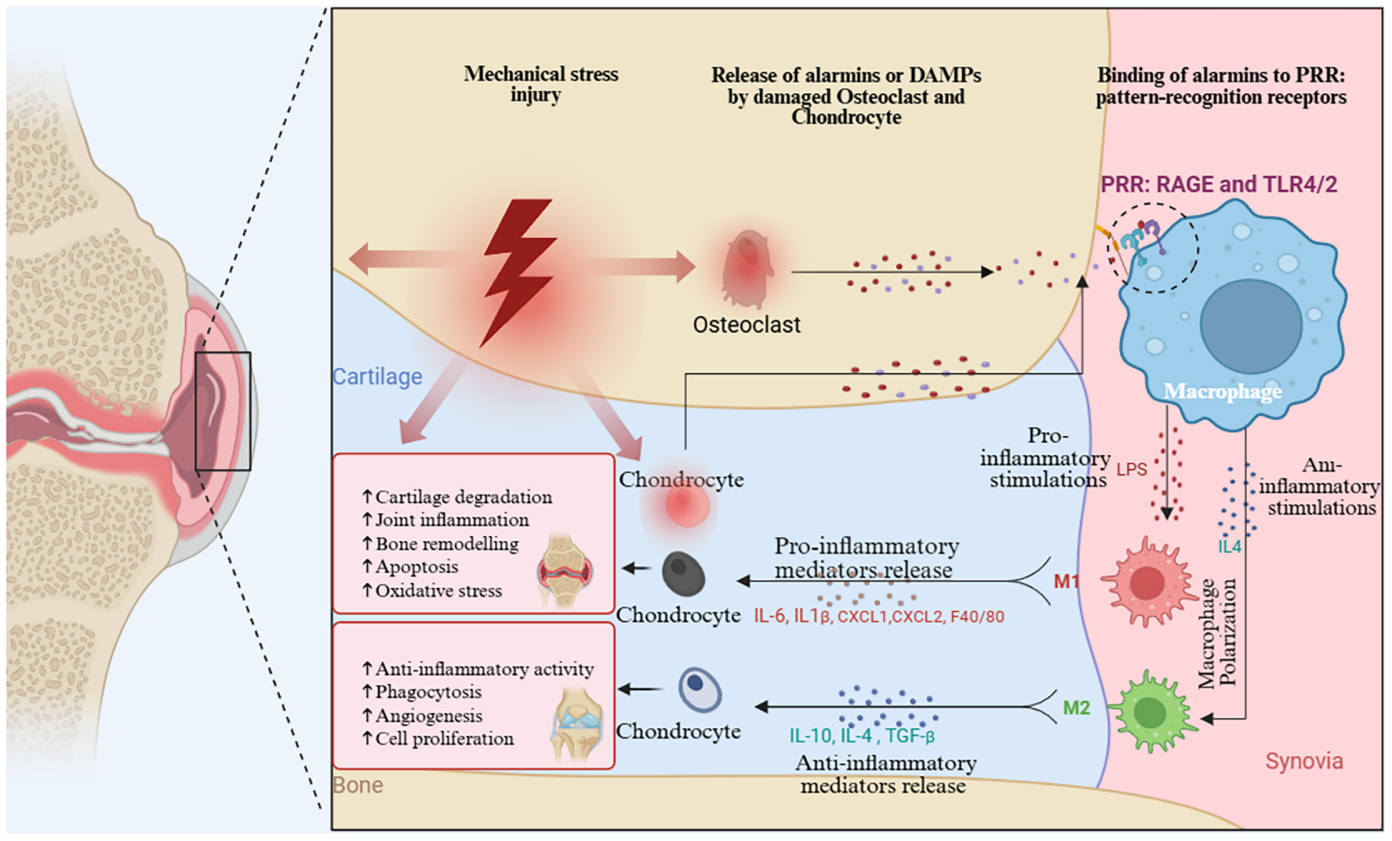
Mechanism of Macrophage Polarization regulating chondrocyte damage caused by OA. (Graphic created in by BioRender (https://BioRender.com).

Our co-occurrence analysis of keywords and research themes highlights several well-established research areas, including macrophage polarization in OA synovium, the role of inflammatory cytokines in cartilage degradation, and macrophage-mediated pain mechanisms. The M1/M2 classification of macrophages has been a useful framework for understanding inflammation in (OA). However, the interaction between macrophages and chondrocytes highlights a more complex regulatory axis in OA pathogenesis **Figure 10**. Recent single-cell RNA sequencing studies have identified at least seven distinct macrophage subpopulations within the OA synovium, each with unique transcriptional profiles and functions. These findings challenge the conventional M1/M2 dichotomy and reveal a diverse spectrum of macrophage phenotypes with overlapping roles (65). Recent studies reveal that macrophages in (OA) can exhibit a hybrid phenotype, expressing both M1 and M2 markers (CD206^+^CD86^+^) and secreting both pro- and anti-inflammatory cytokines. This challenges the traditional M1/M2 model and highlights the complexity of macrophage behavior in OA (42, 66). Macrophage polarization exhibit remarkable tissue-specific functions throughout the joint. study mapped distinct macrophage phenotypes across multiple joint tissues using spatial transcriptomics. This research identified tissue-resident macrophage populations with unique origins and functions (67). Recent findings on the diversity of macrophage polarization in OA done by Nakamura et al. (68). identified infrapatellar fat pad macrophages that secrete adipokines like adiponectin and visfatin, directly affecting cartilage metabolism.

In subchondral bone, Chen et al. (69) described “osteomacs” (F4/80^+^, CD169^+^, MERTK^+^) that regulate bone remodeling and contribute to sclerosis. Also the study discovered meniscus-resident macrophages with regenerative potential, producing matrix components essential for tissue repair (70). The dynamic changes in macrophage behavior across OA stages represent a critical research gap. Longitudinal imaging studies revealed distinct activation waves: early tissue-resident activation, pro-inflammatory macrophage recruitment, emergence of resolution-phase macrophages, and persistence of “failure-to-resolve” macrophages in chronic disease (71). As study showed the targeting these stage-specific macrophage shifts could be key to future OA therapies (72).

Although macrophages are central to OA pathogenesis, effective therapeutic targeting remains challenging. Recent innovations offer promising strategies (73). A recent study developed mannose-decorated liposomes that selectively deliver anti-inflammatory agents to CD206^+^ synovial macrophages, reducing cartilage degradation by 65% in preclinical models. Wilson and Patel et al. introduced small molecule inhibitors that reprogram inflammatory macrophages toward a tissue-repair phenotype by modulating NF-κB and STAT6 signaling (73).

Wong C et al. (74) showed the extracellular vesicle (EV) therapies that from mesenchymal stem cells can shift macrophages to a pro-resolving phenotype in OA joints. Wang J et al. (75), developed biomaterial scaffolds releasing macrophage-modulating factors in a spatially controlled manner to guide repair processes. Moreover, study reported that CCR2 inhibition successfully reduced inflammatory monocyte recruitment while preserving homeostatic macrophages, highlighting a promising translational approach (76). These emerging therapies underscore the need for precision strategies that harness macrophage plasticity to promote joint repair while minimizing chronic inflammation.

### 4.5 Emerging hotspots and future directions

The recent advances in the (OA) studies are devoted to the importance of the molecular mechanisms regulating the polarization of the macrophages and their contribution to the progression of osteoarthritis. The analysis of co-occurrence of keywords related to OA-related macrophage of our study indicates the presence of many new molecular pathways, including mitochondrial dysfunction, endoplasmic reticulum (ER) stress, programmed cell death pathways, including ferroptosis and pyroptosis, and ion channels. These molecular pathways are very interconnected with the polarization of macrophages and they are good therapeutic targets in OA.

#### 4.5.1 Mitochondrial dysfunction and macrophage polarization

Mitochondrial dysfunction is increasingly recognized as a critical regulator of macrophage polarization in OA. Previous study show that mitochondrial metabolism influences the differentiation of macrophages into M1 (pro-inflammatory) and M2 (anti-inflammatory) phenotypes, Mitochondrial dysfunction lead to the excessive production of ROS, which induces an inflammatory environment by promoting the M1 phenotype, exacerbating joint inflammation and cartilage degradation (77). Conversely, mitochondrial bioenergetics in M2 macrophages supports tissue repair and resolution of inflammation (78). In OA, mitochondrial activity is progressively impaired, disrupting chondrocyte energy homeostasis and triggering cartilage destruction. Elevated oxidative stress, mitophagy, and mitochondrial dynamics play key roles in chondrocyte pathology. Moreover, specific mitochondrial DNA haplogroups, such as haplogroup J, have been linked to OA development and progression. Mitochondrial-based therapeutic interventions, including antioxidants and mitophagy modulators, have demonstrated promising effects in alleviating cartilage damage and inflammation caused by OA (79).

#### 4.5 2 Endoplasmic reticulum stress and macrophage polarization

The (ER) plays a crucial role in protein synthesis, folding. When the ER is overwhelmed by the accumulation of misfolded proteins, it triggers ER stress and activating the unfolded protein response (UPR). This response critically regulates macrophage polarization, influencing the shift between M1 and M2 phenotypes which has significant implications in OA.

The UPR is regulated by three key transmembrane sensors: IRE1, PERK, and ATF6. Upon activation, IRE1α splices XBP1 mRNA to produce the active form, XBP1s, which regulates genes involved in protein degradation and ER biogenesis. PERK activation reduces global protein synthesis while promoting selective translation of ATF4 and CHOP, which mediate inflammatory responses. ATF6 is translocated to the Golgi apparatus, where it is cleaved to initiate transcription of UPR target genes, thereby inducing the cell’s adaptive response to ER stress (80). ER stress is a potent inducer of M1 macrophage polarization. The PERK-eIF2α-ATF4-CHOP signaling axis amplifies the expression of pro-inflammatory cytokines such as IL-1β, TNF-α, and IL-6, which contribute to inflammation and cartilage destruction in OA (81). Additionally, ER stress activates NF-κB signaling and the NLRP3 inflammasome, further enhancing M1 polarization and intensifying inflammatory responses (82). Conversely, under certain conditions, ER stress can also promote M2 polarization specially for example chronic ER stress facilitates M2 differentiation by upregulating the expression of arginase-1 (Arg-1), IL-10, and TGF-β, which are markers of alternatively activated macrophages (83). Targeting ER stress pathways for therapeutic intervention in inflammatory diseases such as OA holds promise. Inhibitors like 4-phenylbutyric acid (4-PBA) and Tauro ursodeoxycholic acid (TUDCA) have been shown to promote M2 polarization in experimental models (84).

#### 4.5 3 Ferroptosis and pyroptosis and macrophage polarization

Ferroptosis is an iron-dependent regulated cell death that involves lipid peroxidation, which is implicated in the (OA) development by influencing the macrophages polarization.

In OA, ferroptosis targets chondrocytes, releasing damage-associated molecular patterns (DAMPs) that trigger synovial macrophages. This leads to the polarization of M1 macrophages through toll-like receptors (TLRs) and NF-KB signaling, aggravating inflammation and cartilage destruction (85). Recent studies suggest that ferroptosis can be regulated by microRNA pathways, offering therapeutic potential to control chondrocyte survival and macrophage polarization in OA (86).

Pyroptosis, mediated by the NLRP3 inflammasome, releases IL-1β and IL-18, which driving inflammation in OA. It activates M1 polarization in both chondrocytes and macrophages. Inhibiting NLRP3 or regulating miRNA can shift polarization to M2, reducing inflammation and cartilage damage. Lipoxin A4 (LXA4) has shown potential in protecting chondrocytes and promoting M2 polarization (87). Targeting ferroptosis and pyroptosis with iron chelation, antioxidants, and NLRP3 inhibitors may slow OA progression and enhance joint regeneration.

#### 4.5 4 Ion channels and macrophage polarization

Ion channels such as Nav1.7, TRP, Piezo, and P2X are crucial in OA because they control pain, inflammation, and degradation of cartilage. These channels affect calcium homeostasis, Mechan transduction, and immune cell behavior, with disruptions linked to OA development (88).

Transient receptor potential (TRP) channels, especially TRPV4, influence macrophage polarization in OA. Inhibition of TRPV4 reduces M1 polarization by modulating the ROS/NLRP3 pathway (89). TRP channels, activated by mechanical stress and inflammatory mediators, promote calcium influx that drives M1 polarization and inflammation in OA joints, linking mechanical stress to inflammatory responses in OA (90). Macrophage polarization relies on calcium influx via voltage-gated calcium channels (VGCCs) and store-operated calcium entry (SOCE). Disruptions in calcium homeostasis favor persistent M1 polarization and chronic inflammation in OA. Potassium channels like Kv1.3 and KCa3.1 regulate macrophage migration, cytokine production, and polarization, all critical to OA progression (91). Selectively modulating ion channels to promote M2 polarization and inhibit M1 activation may help reduce inflammation and cartilage degeneration in OA.

Therefore, exploring these ion channels and their role in macrophage polarization could provide insights for developing targeted therapies that alleviate OA symptoms. Further investigation into these molecular mechanisms is necessary.

## 5 Limitation

Some limitations remain to be discussed: (1) We exclusively utilized Scopus and WoSCC, omitting databases such as Embase, which could create publishing bias. Subsequent studies ought to include a greater variety of sources and sophisticated instruments. (2) Only English research and review articles were included, potentially omitting relevant non-English or other articles. (3) Temporal keyword trends were not analyzed, leading to possible bias in predicting research hotspots. (4) Newly published influential research may have been missed due to the continual nature of updating.

## 6 Conclusion

This study offers a comprehensive overview of global research on macrophages in OA over the past decade, highlighting key contributors, journals, and emerging research trends. By utilizing bibliometric and visualization analysis, we identified significant research hotspots such as macrophage polarization, immune modulation, and metabolic reprogramming. These findings underscore the critical role of macrophage-driven inflammation in OA pathogenesis, illustrating the broader implications of immune responses in the disease, and providing insights into novel therapeutic targets. Despite the progress made, challenges remain, particularly the need for standardized research frameworks and greater international collaboration. To address these challenges, future studies should focus on developing standardized protocols for macrophage polarization assessment and uniform criteria for OA animal models, such as consistent methods for induction and monitoring disease progression. Adopting these standardized approaches, supported by consensus guidelines like those from OARSI, will help improve reproducibility and comparability across studies, accelerating progress in understanding and treating OA. Collaboration across disciplines and regions will be essential for translating this knowledge into clinical interventions.

## Data Availability

The raw data supporting the conclusions of this article will be made available by the authors, without undue reservation.
